# Nano-level morphology of scar tissue after myocardial infarction

**DOI:** 10.15190/d.2015.41

**Published:** 2015-09-30

**Authors:** Zhuojun Wu, Adelina Curaj, Mareike Staudt, Victor Ponomariov, Leonardus Decker, Mihaela Rusu

**Affiliations:** Institute for Molecular Cardiovascular Research (IMCAR), RWTH Aachen University, Germany; University of Medicine and Pharmacy, Craiova, Romania

**Keywords:** Atomic force microscopy, scar tissue morphology, nano-fibrils, periodicity

## Abstract

Atomic force microscopy (AFM) is a pioneer imaging technique commonly employed by biological researchers in detection of the properties of biological membranes over the last decade. The AFM findings distinguish its applicability from the conventional methods, such as: confocal, multi-photons, electron microscopy, etc. as well as from the mechanical methods (compression and indentation test, extensiometry, etc.). With its high resolution (below 10 nm), AFM has emerged as a powerful tool in obtaining the nanostructural details and biomechanical properties of heart tissue. The composition of extracellular matrix is essential for heart compliance and its mechanical function. Here, we illustrate the surface morphology, its structural assembling and the mechanical properties of a myocardial infarction scar section aquired via AFM, in dry conditions. The cross section through the mature myocardial scar of mice after myocardial infarction shows that the embedded fibrils into the tissue matrix of a mature scar overlap at some sites, and form network-like structures. The nano-fibrils surface shows defined structural periodicity. A cross-section along the axial fibrilar direction gives an average D-periodic banding pattern of approximately 50,3 nm (± 6,2 nm std.). As future perspective, yet uncovered morphological and mechanical investigations, correlated with functional studies, open a new window for understanding pathological mechanisms.

Atomic force microscopy (AFM) uses a scanning probe microscope based on the interaction of a sharp tip and the underlying atoms of the investigated surface. AFM is a unique research tool to investigate surface properties (friction and adhesion forces, viscoelastic properties, tissue compliance via Young’s modulus, etc.) in three-dimension. The AFM findings will open a wide avenue mostly in the cardiovascular field, where heart function need to be associated with structural changes and functionality. AFM has emerged as a powerful tool to obtain the nanostructural details and mechanical properties of biological samples, including of biomolecules and cells. Remodeling after myocardial infarction starts with a massive extracellular matrix production and deposition as a vectorial mechanism towards fibrosis formation. The cascade of infarction events is reflected in tissue function loss, where the contractile elements start the vicious cycle of mechanical inefficiency and eventual heart failure. Collagen is the main component of the extracellular matrix, providing strength to various structures. The scar tissue formed after an ischemic event is involved in preserving the mechanical structure of the heart, sustaining the pump function of the heart muscle. Scar formation is a dynamic process, starting early after myocardial infarction with synthesis of a fibrin-based matrix. Later, the myofibrobalsts are modulated by numerous factors to synthesize a dense collagen-based scar. Our knowledge with respect to biochemical composition, structure and functionality in the scar tissue at the molecular level is just at the beginning. In myocardial infarction, fibril-forming collagen I and III stabilized the scar, while non-fibrilar collagens IV and VI have an impact on myofibroblasts differentiation and organization of the extracellular matrix network. Furthermore, extracellular matrix proteins, including fibronectin, elastin, laminin, biglycan and other components are required for proper healing of injured myocardial tissue^1-3^. Changes in the two competitive physicochemical parameters of collagen formation and desintegration rates lead to compositional modification of extracellular matrix, which in turn modifies the heart tissue compliance, resulting in a change in heart function^4^. Of a great interest are the nano-assemblies of structural subdomains forming collagen fibrils, which lie between molecular and fibrillar levels. The substructural information relates to the overall mechanical properties of fibrils, by preventing or developing cracks. These cracks may predominantly propagate in the border zone (delineated as the region between scar and remote zones), when undergoing post-myocardial infarction (data not shown). The surface of the fibril is the “mirror” of all interface interactions with adjacent fibrils or other molecules. State of the art morphological techniques, such as AFM, are providing valuable information on fine structural and morphological details, such as molecular heterogeneity^5^. Microscopically, collagenous structures appear as elongated micro-fibrils packed into fibrillar collagen types. Their periodic banding patterns depend on collagen composition and the synthesized sites^6^.

In the **Figure 1**, **(A)** the cross section through the mature myocardial scar of mouse is visualized in a dry state by peak force tapping quantitative nano-mechanics AFM. The embedded fibrils into the tissue matrix of a mature scar overlap at some sites (yellow circles), and form network-like structures (yellow head arrow). Few sites represent the anchoring points (blue head arrows) for some nano-size fibrils, while some fibrils seem to be completely detached (green head arrows). The nano-fibril’s surface shows defined structural periodicity. The nano-fibril’s contour has a well-defined topological profile, while some bundles of fibrils have a faint color. **(B)** The identified periodical structures recorded along the axial direction are depicted with an alternating bright and dark color gradient in the adhesion map. The alternating pattern appears with a spatial distribution of approximately 50 nm. The bright color assigns a low adhesion profile, while the soft nano-segments are illustrated in dark. **(C)** Topographic analysis perpendicular to the longitudinal axis of fibrils suggests a mean fibril height of 20.3 nm (± 4.2 nm std.), whereas a cross-section along the axial fibrilar direction gives an average D-periodic banding pattern of approximately 50,3 nm (± 6,2 nm std.).

Our understanding of the regulatory mechanism involved for fine-tunning the extracellular matrix formation and degradation is fundamental for the development of agents that may serve or prevent excess collagen deposition in cardiovascular diseases.

**Figure 1:**
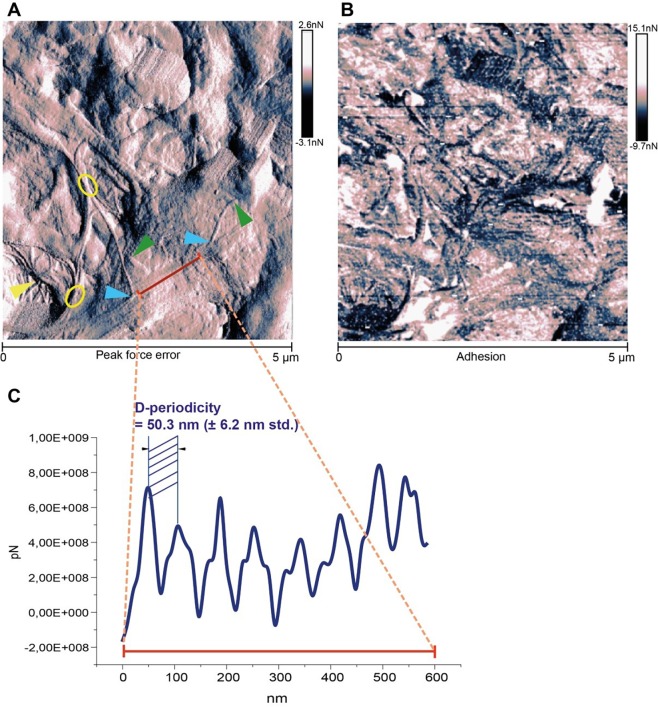
Nano-level morphology of scar tissue after myocardial infarction **(A) **Surface morphology: Peak force tapping quantitative nano-mechanics AFM dry state of the cross section of a mature myocardial scar in mouse heart; *yellow circles*: overlapping fibrils; *yellow head arrow*: network-like structures; *blue head arrows*: anchoring points of nano-fibrils; *green head arrows*: disrupted and detached fibrils. **(B)** adhesion map: surface mechanical properties of nano-fibrils represented by a colour gradient pattern; *bright color*: high adhesion; *dark color*: low adhesion. **(C)** Spectral periodicity of nano-fibrils as performed by off-line section analysis. The average of D-periodicity is 50.3 nm (± 6.2 nm std.).
